# Influence of Context as a Key Ingredient for High Performance on Skilled Nursing Facility Readmissions

**DOI:** 10.1093/geroni/igaa057.128

**Published:** 2020-12-16

**Authors:** Roman Ayele, Kirstin Manges, Chelsea Leonard, Marcie Lee, Emily Galenbeck, Mithu Molla, Cari Levy, Robert Burke

**Affiliations:** 1 VA Eastern Colorado Health Care System, Aurora, Colorado, United States; 2 University of Pennsylvania Perelman School of Medicine, Philadelphia, Pennsylvania, United States; 3 UC Davis Health, Sacramento, California, United States; 4 Center for Health Equity Research and Promotion, Corporal Crescenz VA Medical Center, Philadelphia, Pennsylvania, United States

## Abstract

Improving hospital discharge processes and reducing adverse outcomes post discharge has become a topic of national importance, especially for older patients transitioning to skilled nursing facilities (SNFs). The substantial variation in rehospitalization outcomes suggests contextual factors may play a role in performance. We conducted rapid ethnography with opportunistic interviews (148 hours of observations, 30 clinicians) at hospitals that were high (n=2) or low (n=2)- performing in terms of their readmission rates from SNF, as well as 5 associated SNFs, to understand how contextual differences influenced performance variation. We used thematic analysis to categorize contextual factors and compare differences across high and low perfoming sites. We found three main contextual factors that differed across high- and low-performing hospitals and SNFs: team dynamics, patient characteristics, and organizational context. First, we observed high-quality communication, situational awareness, and shared mental models among team members in high-performing sites. Second, patients at high-performing hospitals had better insurance coverage that made it feasible for clinicians to place patients in SNFs based on their needs instead of financial abilities. Third, staff at high-performing hospitals were more engaged in the transitions process and rapport building with SNFs thereby facilitating smooth transitions of care from hospitals to SNFs. For example, clinicians at high- performing sites were actively engaged in directing the discharge process, but this was not observed in low-performing sites where a lack of clinician engagement lead to delays. Understanding how context affects transitions could be used to target organizational, policy, and staff level interventions to reduce adverse outcomes.

